# Hardening and Fresh State Behaviour of Ternary Cement for Marine Environments: Modification through Nanoadditives

**DOI:** 10.3390/ma15051938

**Published:** 2022-03-05

**Authors:** Amaia Matanza Corro, Céline Perlot, Ema Latapie, Silvina Cerveny

**Affiliations:** 1 Laboratoire des Sciences de l’Ingénieur Appliquées à la Mécanique et au génie Electrique (SIAME), Université de Pau et des Pays de l’Adour, E2S UPPA, 64600 Anglet, France; a.matanza-corro@univ-pau.fr (A.M.C.); ema.latapie@etud.univ-pau.fr (E.L.); 2 Centro de Física de Materiales (CSIC, UPV/EHU)-Materials Physics Center (MPC), 20018 San Sebastian, Spain; celine.perlot@univ-pau.fr; 3 Institut Universitaire de France, 1 rue Descartes, CEDEX 05, 75231 Paris, France; 4 Donostia International Physics Center (DIPC), 20018 San Sebastian, Spain

**Keywords:** nanoadditives, cementitious materials, marine environment, rheology, setting time, calorimetry, mechanical behaviour

## Abstract

The use of nanomaterials to enhance the physical and mechanical properties and durability of cement materials in their hardened state has been studied for a long time in many investigations. In comparison, fewer studies focus on nanomaterials’ influence on the fresh state when the cement reaction starts. In addition, if we consider ternary blended cement (as those used for applications in marine environments), this has been rarely studied. Severe stresses in the marine environment require high durability, which is achieved by using pozzolanic additions, to the detriment of a rapid achievement of the properties. The addition of nanomaterials could contribute to increasing the durability and also accelerating the setting of the concrete. In this work, we performed a systematic and comparative study on the influence of adding graphene oxide (GO), nanosilica (NS), and microfibrillated cellulose (MFC) during the setting mechanisms of cement (CEM V/A suitable for concrete subjected to external attacks in marine environments) blended with fly ash and slag. Cement hardening was examined through setting time and rheology within mini-slump tests. The effect of nanoadditives on the cement hydration was analysed by heat flow calorimetry to evaluate the acceleration potential. Exploring the three nanoadditives on the same formulation, we could establish that the retention of mixing water significantly decreased workability for MFC. In contrast, NS increases the hydration of cement particles, acting as nucleation nodes and promoting supplementary cement hydrates (pozzolanic reactions) and accelerating setting time. Finally, GO showed a reduction in workability. We also investigated the dosage effects on mechanical behaviour at an early age and discovered an improvement even at low GO (0.006%) and NS (3%) dosages. We have also analysed the dosage effects on mechanical behaviour at an early age.

## 1. Introduction

It is well known that a wide range of additives is used to modify the properties of cementitious materials, both in the fresh and hardened state. The primary purpose of this is to optimise the formulation of cement materials to the implementation and use requirements [1,2,3,4,5,6]. In fact, the development of nanoadditives and their contribution to enhancing properties when added to cement-based materials has been the subject of extensive research [7,8,9,10]. Despite this, systematic studies comparing the improvement of properties of different additions in the fresh state and during hardening of cement materials are difficult to find, particularly for ternary cement, which is used in marine environments. In fact, cement mortars for marine exposures improve compressive strength and durability when exposed to marine environments [11,12,13,14]. Chemical aggression coupled with mechanical action in the marine environment is particularly severe for cementitious materials. They require very high durability, which can be achieved using pozzolanic (such as fly ash) and/or hydraulic (such as slag) additions. Pozzolanic reactions consume portlandite, which is a weak element towards durability (sulphate attack, carbonation or leaching). However, these pozzolanic reactions are slow, and the concrete only reaches its maximum properties after 90 days. The use of nanoadditions would allow, on the one hand, to activate the reactions at an early age, but also to promote the development of primary C-S-H, which contribute to durability. Combining these effects for applications in the marine environment seems interesting, although little studied. The hydration degree of cement particles in cement mortars for marine exposures is considered to be fully achieved at 90 days due to the slow reaction of fly ash [15]. Setting accelerators are widely used to control the initial and final setting time by accelerating the hydration process of grouts, mortars and concretes [16]. This is particularly important in cold weather areas or even for removing and reusing formwork on-site at faster rates, or in marine construction to comply with the tide. However, this type of admixture brings other problems to concrete, such as surface cracking due to autogenous shrinkage [17]. In addition, the modification of setting time could affect the workability of the mixture.

In the current context of the energy transition, nanomaterials have been explored to reduce the environmental impact of cementitious materials [18]. The improvement of concrete performance minimises the environmental impact [19]. Furthermore, reusing or recycling by-products to develop nanoadditives reduces the concrete carbon footprint, considering CO_2_ emissions in the life cycle assessment [19]. The incorporation of a relatively wide variety of nanoadditives of different sizes and shapes into cementitious materials to improve their rheological and mechanical properties has been studied in the literature [19,20,21]. Nanomaterials can interact with cement particles to modify their hydration in terms of kinetics or even products formed [21]. NS acts as a nucleation node and promotes the formation of hydration products due to their high specific surface and reactivity [22]. They can also interact with the mixing water, reducing the available water to react with cement particles and modifying the rheology or the setting. Even with low amounts, the benefits are observed from the beginning of the cement hydration reaction [23]. However, the key of optimal enhancement is related to achieving an effective dispersion [20] as indeed nanomaterials tend to aggregate forming clusters due to solid attraction forces at the nanoscale. Unreacted agglomerations could become weak points and result in bad mechanical performance [24]. Ultra-sonication and superplasticisers are, therefore, essential to obtain a suitable performance even at low loadings [25]. As previously mentioned, we will present in this investigation a systematic study using the three different nanoadditives regular in the cement industry (graphene oxide (GO), micro fibrillated cellulose (MFC) and nanosilica (NS)). We investigated the influence of different nanoadditives contents on the fresh state performances of cementitious materials in order to develop a comparative difference between them.

Graphene oxide nanoplatelets (GO) are produced by the thermal or mechanical exfoliation of functionalised graphite using sonication [26]. After this process, the GO is chemically purified to obtain a hydrophilic dispersion of mostly monolayer nanoplatelets. Due to its particularly low electrical conductivity, GO is widely used for electronic applications. GO can be easily dispersed in polar solvents, like water, due to the presence of carboxyl and hydroxyl groups. This particular chemical configuration makes GO an attractive alternative to carbon nanotubes, which are hydrophobic and more difficult to disperse in water. This hydrophilic property allows a good spreading of GO in the cementitious matrix. Most of the previous studies of GO indicate that it acts as extra sites for cement hydrates that grow on the surface of the nanoplatelets [27,28,29], although Ghazizadeh [30] reported that is linked to the presence of gypsum. Additionally, the hydroxyl groups increase the chemical reactivity with cement particles [31]. As a consequence, the addition of GO improves the hydration degree on cementitious materials [32]. Apart from mechanical enhancement, durability related properties are also improved due to porosity reduction [33]. The pore’s size and its connectivity are reduced, hindering the diffusion of harmful compounds into concrete [34]. Therefore, GO can be considered as an excellent candidate to be used as an additive for cementitious materials [35] as far as an optimal dispersion within the matrix can be easily achieved [36].

Cellulose is the main component of the plant cell wall consisting of a very long linear chain of D-glucose units. Each cellulose molecule is rod-shaped and contains numerous hydroxyls groups. The hydrogen bonds between adjacent chains let them pack closely into cellulose fibres. Another way to improve cement hydration is to incorporate nanocellulose in microfibres (microfibrillated cellulose, MFC) produced by the mechanical refining and chemical purification of wood or plant fibres [37]. To obtain the microfibres, cellulose is treated with a high-pressure homogeniser and a super micro-grinder [38]. Since these fibres can be obtained from renewable vegetable sources, they have a relatively low cost. Therefore, the great advantage of MFC compared to other nanomaterials like GO is that it is an environmentally friendly material. As MFC acts as a water reservoir, it has a significant impact on the rheology of nanocellulose reinforced cementitious materials due to the hydrophilic qualities of the fibres. MFC tends to retain water throughout the mixing process, lowering workability in the fresh stage [39]. In the long term, MFC stores water that can be used for further hydration [11] and reduce the shrinkage if enough external water source is provided during concrete curing or use [40].

Nanosilica (NS) is a white powder containing amorphous SiO_2_ with typical diameters well below 100 nm. Due to its large specific surface area and silica content, NS plays a significant role as a pozzolanic activator [6]. A small quantity of NS in the cement paste accelerates the dissolution of tri-calcium silicate (C_3_S). Therefore, a faster formation of the C-S-H phase is produced during cement hydration [41]. Moreover, NS consumes free calcium hydroxide to form additional C-S-H [42] and reduces calcium leaching [42]. The hydroxyl groups on its surface allow a good dispersion in water, improved by the addition of superplasticisers which prevent agglomeration and workability reduction [5]. Thus, NS improves the mechanical properties due to a densification of the microstructure and refinement of the pore structure of the cement matrix [43,44]. The optimal amount of NS depends on the water/cement ratio and the superplasticiser dosage [44,45].

The present study was carried out to identify the changes in the setting mechanisms of ternary blended cement for marine structures when different dosages of GO, MFC, and NS are added. The cement selected for this research is suitable for marine environment applications where exposure to climate conditions will determine the infrastructure’s durability. Typically, concrete infrastructures can be exposed to many chloride ions up to 5 km from the coastline. Chloride and carbonate ions will permeate the porous structure of the concrete. Following that, degradation will start once the corrosion threshold is achieved near the reinforcing steel bars. Furthermore, chloride ions can combine with C_3_A and C_4_AF hydration products to create Friedel’s salt, resulting in faster corrosion failure. To prevent chloride binding in such harsh conditions, the type of cement is selected depending on mineral composition. The amount of C_3_A is limited for seawater resistant cement types. For cement type V, C_3_A should be below 8% and the content of C_3_A + C_4_AF, below 25%. Our focus in this study was investigating the fresh state and relating these properties with the hardened state’s properties via mechanical characterisation. Therefore, modifications on the hardening of blended cements through setting and calorimetry tests were characterised. Rheology studies were conducted with mini-slump tests. Finally, mechanical properties were investigated using measurements of compressive and flexural strengths.

## 2. Materials and Methods

### 2.1. Nanomaterials

The nanoadditives used in this study were selected according to industrial availability and ability to disperse. Three different nanomaterials were considered because of their different chemical compositions and morphologies: GO nanoplatelets, MFC microfibres and NS nanoparticles. Thus, the influence of these two aspects on cementitious materials properties could be observed.

#### 2.1.1. Graphene Oxide (GO)

Graphene oxide is a bi-dimensional and non-stoichiometric chemical compound of carbon, oxygen, and hydrogen in variable ratios. GO from Graphenea^®^ (San Sebastian, Spain) used for this study was a dispersion of graphene oxide sheets in water at the concentration of 4 mg/mL. GO flakes are monolayers of 2 nm thickness (determined via AFM) in dilute dispersions of 90% of monolayers flakes (nanoplatelets) with an average size up to 10 µm. If diluted to 0.5 mg/mL and sonicated repeatedly, the monolayer content may be increased to 95%. The elemental composition of GO is detailed in Table 1.

#### 2.1.2. Microfibrillated Cellulose (MFC)

Cellulose is a natural organic material composed of carbon, oxygen and hydrogen (carbohydrate). Microfibrillated cellulose was supplied by Exilva© (Borregaard, Sarpsborg, Norway). provided in slurry form with a solid concentration of 2% (2 g of microfibrils and 98 g of water in 100 g of suspension). MFC has a nominal fibre width of 50 nm and a length of up to several hundred micrometres. Due to their high surface area and surface hydroxyl groups, MFC is considered hydrophilic. MFC characteristics are detailed in Table 1.

#### 2.1.3. Nanosilica (NS)

Nanosilica used in this work is a nanostructured amorphous silica powder of 140 m^2^/g surface area with an average size of 15 nm. Commercialised as Nyasil 20, it was provided by Nyacol^®^ (commercialised as Nyasil 20) (Nyacol^®^ Nano Technologies, Inc., Ashland, MA, USA). NS characteristics are shown in Table 1.

### 2.2. Cement

The cement used in this study was CEM V/A, named according to the standard NF EN 197-1, and provided by Calcia^®^, (HeidelbergCement Group, Airvault, France). This ternary blended cement comprises 56% clinker, 22% blast furnace slag and 22% fly ash. As pozzolanic and hydraulic materials enhance durability, CEM V/A will be suitable for concrete subjected to external attacks in marine environment. The mineral composition and characteristics of this cement are detailed in Table 1.

### 2.3. Aggregates

For mortar samples, siliceous standard sand 0/2 mm was chosen, provided by S.N.L© (Société Nouvelle du Littoral, Leucate, France). It is reputed to have no water absorption.

### 2.4. Superplasticiser (SP)

The third-generation type superplasticiser with a polycarboxylic ether (PCE) formulation guarantees good workability to the cementitious materials. According to the literature, nanoparticles tend to agglomerate because of electrostatic or chemical interactions. However, utilising a superplasticiser results in excellent dispersion due to steric repulsion [31]. For this reason, a PCE superplasticiser (EthacrylTM HF, diluted in water with 40% solid content) was selected for this study.

### 2.5. Samples Formulation Design

The first objective of this work was to understand the effects of nanomaterials on hydration reactions, which take place in the cementitious phases. For that purpose, the hardening study through setting time and calorimetry was performed on cement pastes. Secondly, to observe the influence of nanoadditives on rheology and mechanical behaviour of cementitious materials (i.e., aggregates surrounded by cement paste), sand was added to the cement pastes to lead tests on mortars.

Preliminary studies were conducted to determine the mix ratio range for each nanoadditives. Several samples were manufactured with different contents and mix ratio ranges were fixed from compressive test results. Finally, 4 contents were considered for GO: 0.006%, 0.09%, 0.03% and 0.1% and samples are named according to these contents, respectively, as GO 0.006, GO 0.009, GO 0.03 and GO 0.1. MFC and NS were prepared in 3 different concentrations. For MFC, 0.06%, 0.09% and 0.15% were used and consecutively named the samples as MFC 0.06, MFC 0.09 and MFC 0.15, respectively. For NS, on the other hand, 2%, 3% and 4% were added and the samples were named as NS 2, NS 3 and NS 4, respectively. The amount of nanoadditives is given by the percentage of cement mass. The reference formulation (REF) keeps the same water/cement and superplasticiser/cement ratios as the nanoreinforced ones.

The W/C ratio was 0.4 for all the formulations. This value was chosen according to the exposure classes in the standard NF EN 206-1, as this study was part of a larger project oriented to concrete durability improvement of infrastructures in a marine environment. Since nanomaterials were supplied in slurry or pre-dispersed into an aqueous solution, mixing water amounts were corrected to keep a constant W/C ratio. From previous studies, the optimal PCE superplasticiser amount was determined as 0.6% of cement mass. All cement paste formulations studied are described in Table 2.

For mortar samples used in mechanical tests, a sand/cement ratio of 3/1 is adopted, which corresponds to 1350 g of sand for 450 g of cement. Same formulations as cement paste samples are used, adding standard normalised sand to the formulations described in Table 2 to obtain mortars.

### 2.6. Samples Preparation

#### 2.6.1. Nanoadditives Solution

Chemical and electric interactions between nanomaterials tend to agglomerate the particles or fibres in clusters. An optimal dispersion in cement matrix is critical to fully develop their properties and achieve maximal performances [31]. When using hydrophobic nanomaterials, the dispersion must be done with the help of a superplasticiser. If the nanomaterials are hydrophilic, the dispersion can be done in the mixing water. Ultra-sonication of nanoadditives solutions is the best method to split the clusters completely, obtain optimal dispersion, and prevent the additions caking. In addition, using a superplasticiser will allow keeping the nanomaterials in the solution for a longer time: it can be stored and reused later with a short ultra-sonication mix.

Both GO and MFC were prepared pre-dispersed in water solution or slurry form by the supplier. They were manually dispersed in mixing water and superplasticiser, correcting the amount of added water to take into account water content in nanoadditives solutions (to keep W/C ratio of 0.4). Ultrasonication for 10 min was applied to achieve an accurate distribution of the nanoadditives solution. This solution was prepared at different concentrations of nanoadditives for all nanomaterials considered here. NS powder is mixed with cement before adding it to the mixer.

#### 2.6.2. Samples for Setting Time Test

Cement paste samples were prepared according to NF EN 196-3 standard [46] to determine the setting time with a Vicat needle. These cement paste samples did not have the composition described in Table 2. The formulation was specific to this test and is referred as “standard consistency”. The formulation for standard consistency must be determined for each mix composition to be compared, following the procedure: (1) Mix of nanoadditives solution and cement (initial W/C ratio was 0.26) during 90 s at low rate + stop 15 s + mix 90 s, (2) pour the paste into the Vicat’s mould, (3) place the consistency probe of the Vicat’s apparatus at the surface of the paste and release: If the probe reaches 6 ± 1 mm from the bottom of the mould, the standard consistency is achieved. Otherwise, the W/C ratio is decreased keeping superplasticiser/cement ratio constant until the target value is attained. For the REF samples, GO 0.006 and GO 0.009, the standard consistency was achieved for W/C = 0.20, and for the others samples for W/C = 0.21.

#### 2.6.3. Samples for Isothermal Calorimetry

The hydration heat value is measured using 5 g of cement paste directly prepared into the glass vials following the procedure: (1) weigh the required amount of cement in the vial, (2) add the nanoadditives solution to the cement into the ampoule, (3) homogenise the paste using a vortex external mixer (800 rpm during 90 s), (4) stop the mixing for 60 s, (5) mix the paste for 90 s. This procedure was adopted to reproduce the samples’ preparation like in the other tests performed in this study and ensure that all the cement was thoroughly wet with the nanoadditives solution. Note that the masses of cement and compounds of the liquid phase were adjusted to provide 5 g of sample, according to the formulations detailed in Table 2. For heat flow measurement, 5 g of distilled water were used as a reference.

#### 2.6.4. Mortars

The nanoadditives solutions were prepared by mixing the nanomaterials within superplasticiser by ultrasonication as described above. Then, the mortar samples were manufactured according to the procedure from the standard NF EN 196-1 [47] in a conventional mortar mixer, according to this recipe: (1) mix cement and sand for 30 s, (2) add the mixing water with the nanoadditives solution and mix at low speed for 60 s, (3) stop the mixer for 60 s to scrape the wall and the bottom of the mixing bowl, (4) mix the mortar for 90 s more at high speed. After mixing, fresh mortars were poured in two layers into 40 × 40 × 160 mm^3^ metallic mould. Each layer was compacted using a jolting table. Samples were kept 24 h in sealed plastic bags before demoulding. Some samples were directly tested, and the remaining were preserved in water until testing.

### 2.7. Testing Methods

#### 2.7.1. Setting Time Measurements

Setting time corresponds to the beginning of cement paste hardening, and it is used both as an indicator of hardening and workability. The setting time was obtained using the Vicat’s needle test under the standard NF EN 196-3 [46]. The tests were performed on cement pastes samples at the same consistency (see above) just after mixing (t_0_). The Vicat’s needle was placed at the surface of the sample and released every 10 min. The initial setting time (t_1_, min) was defined when the penetration depth (d, mm) reached 4 ± 1 mm from the surface. Then, the mould was reversed, and a needle with a circular ring was placed at the surface of the mix and hooked to the Vicat apparatus. The final setting time noted t_2_ (min) was obtained when the circular ring failed to penetrate the paste and did not leave a complete circular impression on the paste surface. Each formulation was tested three times.

#### 2.7.2. Isothermal Calorimetry Tests

The isothermal calorimetry test allows studying the kinetics of hydration reaction of cement and the amount of cementitious formed products through the measurements of the heat flow released during the cement hydration. It can be used to calculate the heat of hydration of cement paste samples as specified in European Standard NF EN 196-11 [48]. The heat flow considered is the difference between the heat flows of the tested sample (a vial with 5 g of cement paste) and a reference sample (a vial filled with 5 g of distilled water) to eliminate the calorific effects of water. The tests were performed in a TAM Air calorimeter of TA Instruments^®^ (New Castle, DE, USA) calibrated at 25 °C. The results were normalised to the sample mass to be compared. Hydration was then followed by isothermal calorimetry for 40 h.

#### 2.7.3. Workability at Fresh State: Mini-Slump Test

Fresh state behaviour of nanoadditives reinforced mortars was evaluated through the mini-slump test method using a mini-cone test, a smaller version of Abram’s cone. The parameters of this test have been discussed by Roussel et al. [49]. This test enables the workability study and its results the calculation of plastic yield stress τ_0_ (Pa) as a rheology indicator:

(1) $$\tau_{0}=\frac{225{\;\rho \;g\;V}^{2}}{128{\;\pi }^{2}{\;R}^{5}}-\;\lambda \frac{{\pi \;R}^{2}}{V}$$

where ρ is the studied fluid volumetric weight (kg/cm^3^), V the tested sample volume (considered as the cone volume, in cm^3^), and R the final spread radius (cm). λ constant takes into account the influence of the unknown fluid surface tension and contact angle. Roussel et al. [49] assumed that this coefficient is the same for any cementitious material for a given test surface and it can be obtained by fitting the predicted spread to the measured spread. In this study, the spread diameter did not reach more than 35 cm. Therefore, it was considered negligible, and the second member of Equation (1) was equal to zero.

The dimensions of the brass mould used in this study were 15 cm in height, the top opening diameter of 5 cm, and the bottom opening diameter of 10 cm. The slump was measured at different durations: 0 (just after mixing), 30, 60 and 90 min to observe the evolution within time and the influence of nanomaterials. For preventing desiccation, mixtures were conserved in a closed batch. Before each slump test, they were mixed for 10 s at low speed in a mortar mixer.

The fresh cement paste was poured in a 1 L volume recipient, and the mass was weighted to obtain the density used in Equation (1). The density of the reference sample was 2016.7 kg/cm^3^. For all the formulations, the density of the fresh state cement paste increased with the addition of GO, by 7% to 8% (GO 0.006 2163.3 kg/cm^3^; GO 0.009 2176.7 kg/cm^3^; GO 0.03 2164.0 kg/cm^3^; GO 2163.7 kg/cm^3^). All the samples with MFC reduced the density by 10%, 8% and 6%, respectively (MFC 0.06 1810.9 kg/cm^3^; MFC 0.09 1843.9 kg/cm^3^; MFC 0.15 1890.9 kg/cm^3^). On the other hand, the sample with 2% NS kept a value similar to the reference one (2024.5 kg/cm^3^). For NS formulations, the samples with 3% and 4% decreased density value by 4% compared to the reference.

To minimise the experimental protocol variation, for each formulation, the mini-slump test was carried out on three replicates from individually mixed batches to obtain an average value of the spread size measured on two diameters for each sample. To minimise experimental protocol variations, a rigorous procedure was used to maintain the speed of removing the mould, the time at which the measurement of the test outcome was taken, the surface on which the mini-slump testing was performed, and the aspect ratio of the mini-slump test mould [49].

### 2.8. Mechanical Tests

To investigate how modifications in fresh properties relate to hardened properties, compressive and flexural tests on mortar samples were performed to evaluate mechanical properties through hour time, according to NF EN 196-1 standard [47] at 24 h ± 15 min and 7 days ± 2 h. For each formulation, flexural tests were conducted on three specimens and compressive tests on six specimens.

## 3. Results and Discussion

### 3.1. Modifications of Setting Time

**Figure 1:**
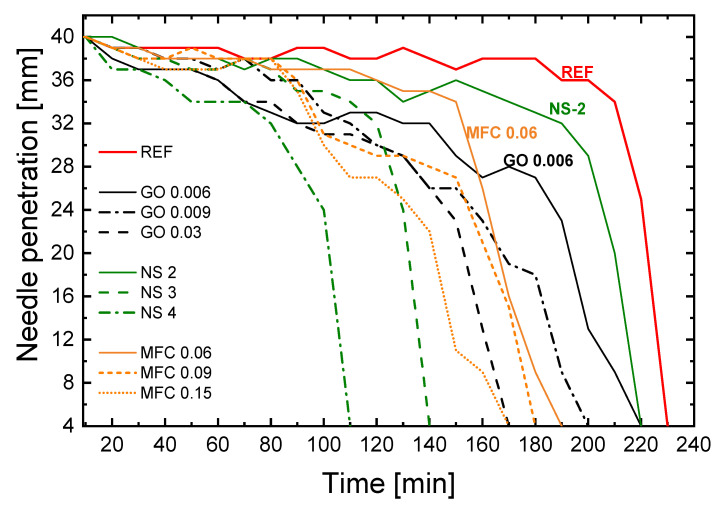
Setting time: evolution of Vicat’s needle penetration depth as a function of time for the different nanoadditives nature and content.

The penetration depth of the Vicat needle with time shows the influence of nanoadditives, nature and content, on setting time (Figure 1).

**Figure 2:**
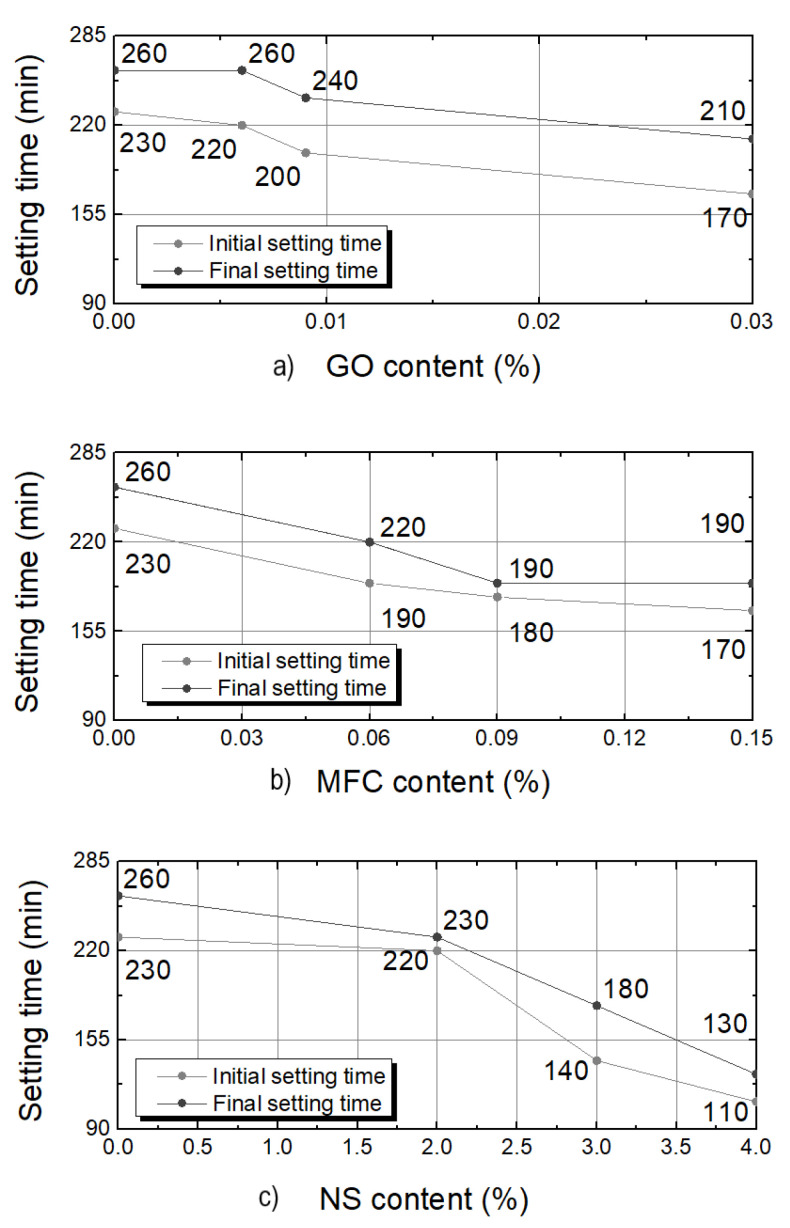
Initial and final setting times for different contents of GO (**a**), MFC (**b**) and NS (**c**). Error value: 10 min for all the measurements.

The curves of nanoadditives reinforced formulations compared with the reference cement paste (REF) indicated that all additions reduced the setting time. The effect appeared to be more marked in the presence of NS. Figure 2 shows the values of initial and final setting time as a function of nanoadditives content for (a) GO, (b) MFC, and (c) NS.

For GO, compared to the reference, initial setting time decreased about 4%, 13% and 26% for 0.006%, 0.009% and 0.03% of GO addition, respectively. The decrease of the setting time was due to GO promoting cement hardening reactions and likely because it acts as nucleation nodes. The final setting time was not modified for GO 0.006 sample. For GO 0.009 and GO 0.03, the final setting time is reduced by 7.7% and 19.2%.

Concerning MFC addition, the initial setting time decreased by about 17%, 22% and 26% for, respectively, 0.06%, 0.09% and 0.15% of content. As soon as a small quantity of MFC is added, the setting time decreases by 17%, but by adding more MFC, the decrease is not so significant. This suggests a saturation effect occurred. MFC enhances chemical reactions by providing nucleation sites. Still, the fibrillated aspect of this nano-additive likely limits the nucleation effect (MFC has a lower specific surface area than GO flakes). The increase of the MFC concentration has only a minimal impact once MFC covers the external surface area of cement grains. Cao et al. [21] explained that the nanocellulose fibres adhered to the cement particles will work as water paths through the shells formed by the hydration products around the unhydrated cement particle. These paths will provide water to promote the hydration of the inner unhydrated cement core. However, beyond this physical aspect, MFC also had a chemical effect. MFC is an organic and hydrophilic compound, and, as described in [35], saturated MFC surrounded cement particles accelerate the cement hydration. Combining these two effects could accelerate the setting more significantly than with the presence of GO, as the initial setting time is reduced from 260 min for REF to 210 min for GO and 190 min for MFC. In relation to the final setting time, the reduction was 15.4% for 0.06% of MFC addition. For both 0.09% and 0.15% additions, the reduction of the final setting time was 26.9%. As observed for the initial setting time, there was a saturation effect.

For NS, the initial setting time decreased slightly for 2% of NS only (4.3%), whereas the decrease was huge for higher contents: 39.1% and 52.2% for, respectively, 3% and 4% of NS addition. The relative reduction of the setting time was more significant for contents from 3%, which proved a threshold level at which particles had an optimal effect. The final setting time was reduced by 11.5%, 30.8% and 50% for the NS 2, NS 3 and NS 4 samples, respectively. NS addition induced a faster setting of cement pastes than GO and MFC addition. This could be attributed both to the morphology and to the chemical composition of NS particles [44]. GO is mainly composed of carbon and enhanced the development of cement hydrates by acting as a nucleation node [32], although other authors disagree with this [30,50]. Since the NS particles are about ten times smaller than GO flakes and show a high specific surface area, the nucleation effect was more intense. In addition, silica constitutes more than 30% of the mineral phases of cement and it is one of the main constituents of cement hydrates, calcium silica hydrates (C-S-H) [51]. For this reason, in addition to the nucleation effect, NS promoted the germination of cement hydrates.

The time-lapse between initial and final setting times for the reference sample, was 30 min. For GO samples, this time-lapse is of 30 min. Any amount of GO did not modify this duration. In contrast, MFC and NS additions reduced this time-lapse significantly. The time-lapse for MFC samples is reduced to 10 min adding 0.09% of nanocellulose. This reduction is more marked for NS, as will be explored further in the next section.

### 3.2. Influence of Nanoadditives on Isothermal Calorimetry

**Figure 3:**
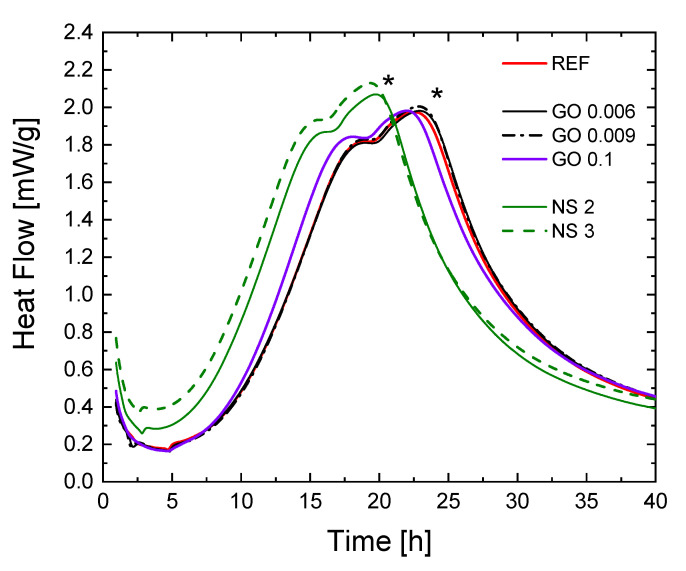
Hydration curve: evolution of heat flow with time for GO and NS additions.

The heat flow released by the cement pastes during the first 40 h of hydration was measured by isothermal calorimetry (Figure 3) for samples with GO and NS additions (measurements could not be carried out on pastes with the addition of MFC). The relative heat flow evolution with time (hydration curve) is divided into four phases, describing the hydration phenomenon during time: initial reactions, induction, accelerating and decelerating periods [52]. Two peaks are observed in the hydration curve. The first one is linked to the tricalcium silicate (C_3_S) reaction. The second peak, related to sulphate depletion is marked with a “*” in Figure 3, when the tricalcium aluminate (C_3_A) hydration starts and produces ettringite (AFt). AFt contributes to the strength of cementitious materials at an early age, and is associated with the early setting with high heat release.

The hydration curves of samples with GO and NS have the same functional form compared to the reference. This tends to show that the presence of the nano-particles did not modify the nature of hydration products. The hydration curves for GO 0.006 and GO 0.009 are similar to the reference one. Therefore, it is assumed that the amounts of GO were too low to modify hydration reactions for these dosages. By contrast, NS have two distinctive effects on heat flow. On the one hand, NS significantly increased the heat emitted over the first 20 h, indicating that more hydrates were generated. On the other hand, the position of the peaks shifted towards shorter durations, reflecting the kinetic catalytic effect of NS. The characteristics of the isothermal calorimetry tests are collected in Table 3. We display the minimal heat flow reached during the induction period (mW/g), the duration of initiation and induction periods (h), the time when sulphate depletion occurred (h) and the heat flow at this moment (mW/g). Cumulative heat (J/g) represented the total hydration heat released over 6 days.

The data in Table 3 established that NS addition has a more substantial influence than GO on cement hydration at an early age. Indeed, NS addition reduces the induction period, and increased heat by about 64% and 124%, respectively, for NS 2 and NS 3. This difference in favour of NS is because SiO_2_ triggers the hydration more efficiently than GO [45], since it can react readily with the cement components and acts as new nucleation sites [44]. In agreement with this, it is observed that the induction period and acceleration period consequently had been shortened for NS. This result agreed with the acceleration of cement hydration observed in the setting time test for NS.

The heat flow peak was reached 15% (NS 2) and 16% (NS 3) earlier, but the heat variation with the reference was not significant: about 5% for NS 2 and 7% for NS 3. This may be due to smaller particle sizes that allowed a rapid increase in nanomaterials surface areas, resulting in a rapid rise in nucleation nodes. These sites were highly active and unstable at early ages, resulting in a faster reaction speed of cement hydration and consequently a higher hydration heat. There was a significant release of heat at the beginning of hydration, which was less and less important over time. This slowing down was mainly due to the reduction of reactive surfaces. Moreover, the cement used in this study is composed of blast furnace slag. The supplementary cementitious materials increase the heat of hydration due to the filler effect and an additional contribution related to their participation in the hydration reactions.

### 3.3. Workability Changes with Nanoadditives

**Figure 4:**
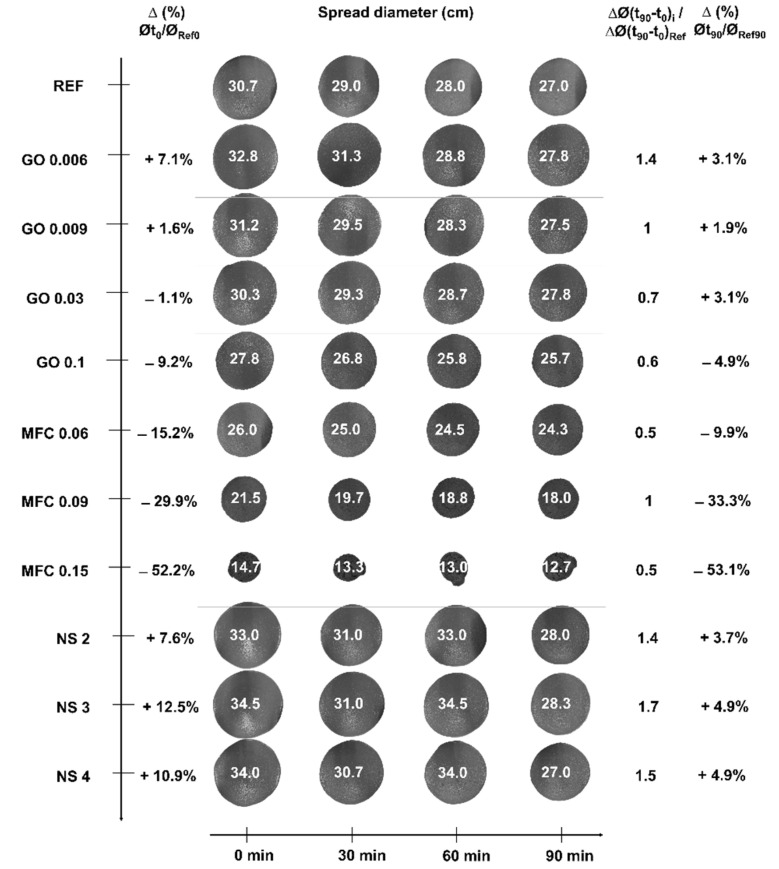
Influence of nanoadditives’ nature and content on mortars spread with time. Error values on spread diameter at 0, 30, 60 and 90 min: Reference samples: 0.58, 0, 0, 0; GO 0.006%: 0.29, 0.29, 0.29, 0.29; GO 0.009%: 0.29, 0.50, 0.29, 0; GO 0.03%: 0.58, 0.58, 0.76, 0.58; GO 0.1%: 1.04, 1.04, 1.04, 1.15; MFC 0.06%: 0.87, 0.87, 0.87, 1.15; MFC 0.09%: 0.87, 1.04, 0.76, 0; MFC 0.15%: 0.29, 0.58, 1, 1.15; NS 2%: 0.87, 0, 0.58, 0; NS 3%: 0.5, 0, 0.58, 0.29; NS 4%: 0.87, 1.15, 0.87, 1.15.

In Figure 4, the pictures of the spread with time present the influence of nanoadditives on mortars workability within a reference grid to compare all the formulations. Pictures were taken at the same time lapse 0, 30, 60 and 90 min after mixing, and from the same distance to be compared. On the left, the variation ∆ (%) of the initial spread diameter Øt_0_ for the mortar with nanoadditives is related to the initial reference spread diameter value at 0 min, Ø_Ref0_. On the right, ∆Ø (t_90_-t_0_)/∆Ø (t_90_-t_0_)_Ref_ represents the spread diameter variation for 90 min for the mortars with nanoadditives divided by the variation of the reference mortar. The reduction ∆ (%) on the spread diameter at 90 min of the reinforced mortars Øt_90_ compared to the reference value at 90 min Øt_Ref90_ is indicated in the last column.

For GO and NS, the effect on the workability is aligned to the performance of the reference sample. Concerning GO addition, the same trends were observed for the same testing time: the addition of low GO contents (0.006 and 0.009) slightly favoured the spreading, whereas higher amounts slightly limited it. As the calculated parameter ∆Ø (t_90_-t_0_)/∆Ø (t_90_-t_0_)_Ref_ is over 1 (for GO contents of 0.006% and 0.009%), it means the GO increases the workability of the mortar at 90 min. For higher amounts of GO, GO 0.03% and GO 0.1%, this value is below 1, because the workability of the mortar at 90 min is reduced compared to the reference sample.

Finally, at 90 min, spreads of mortars with or without GO were in the same order of magnitude. The effect of increasing the quantity of GO added to the spreading diameter should be emphasised. In low contents of 0.006% and 0.009%, the spread diameter increased by 7.1% and 1.6% compared to the reference formulation. The effect is the opposite for higher contents, 0.03% and 0.1%, decreasing the spread diameter by 1.1% and 9.2%. The index calculated to assess the total change on the spread diameter between 0 and 90 min on nanoadditives formulations to reference one demonstrates that rheology rises at low contents, but does not influence at larger contents.

Regarding MCF, the spread diameter was noticeably reduced with the addition of microfibrils, correlated to the nanomaterials content: initial workability was reduced by 15.2%, 29.9% and 52.2% using 0.06%, 0.09% and 0.15% of MFC addition, respectively. After 90 min, the diameter continued to be reduced by 9.9%, 33.3% and 53.1%. Just after mixing (t_0_), the addition of NS increased the initial workability by around 7.6%, 12.5% and 12.9% for, respectively, 2%, 3% and 4% of NS addition. The rheology improved with NS, as shown by the index higher than 1 for all samples. This behaviour seems initially slightly antagonistic with the strong reduction of the setting time measured before. Further analysis of the rheology parameters, particularly yield stress, is necessary. For the later testing times, the workability was barely affected.

On the other hand, the results for MFC were completely different than for GO and NS. The workability is highly reduced with the addition of MFC, and the effect is important with the increasing amount of this nanomaterial. The results suggest that a part of the mixing water is kept by the nanocellulose fibres and causes a reduction of the water available [39]. MFC particles hold back this water and work as a “reservoir” to release water at later ages, promoting the further hydration of cement particles, consistent with the modification of the setting time, Section 3.1. Indeed, MFC is hydrophilic and has the capacity to capture water: MFC could act as a curing agent. However, the effect at early ages is demonstrated to be an important reduction of workability due to the water kept in cellulose microfibres.

Regarding the evolution of the workability with time, from the ∆Ø (t_0_-t_90_)_i_/∆Ø (t_0_-t_90_)_Ref_ factor in Figure 4, values greater than 1 mean that the additions contribute to maintaining the workability over time. In the case of the GO samples, this factor decreased with the increase of GO dosage. As observed for the isothermal calorimetry, there was an acceleration of the cement hydration reaction with a higher amount of GO. With more available nanoplatelets acting as nucleation nodes, the reaction is accelerated. This was consistent with the results that were obtained for the setting time and isothermal calorimetry discussed above. For MFC samples, this ratio varied between 0.5 for MFC 0.15% and MFC 0.06%, and 1 for MFC 0.09%. On the one hand, the results of setting time testing showed that the cellulose fibres accelerate the setting. However, more importantly, the chemical nature and morphology of MFC are made to serve as water-absorbent, which in consequence has a strong impact on workability. The significant workability improvement observed at t_0_ for NS is maintained over time compared to the reference sample.

**Figure 5:**
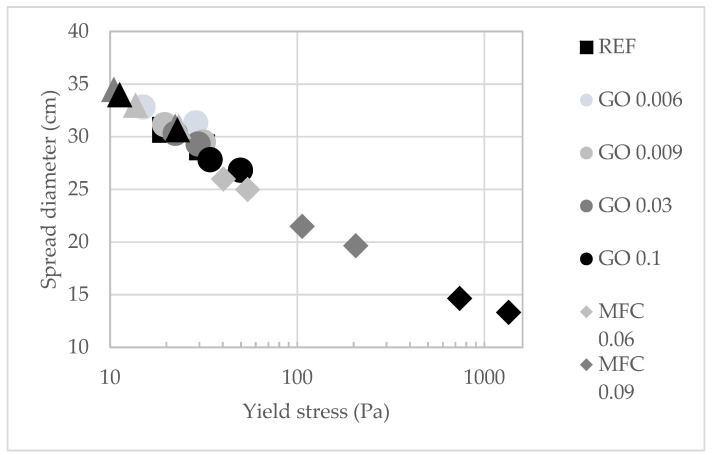
Evolution of mortars’ spread diameter with yield stress as function of nanoadditives nature and content.

To analyse mortars rheology, yield stress τ_0_ was calculated from the Equation (1). The evolution of the spread diameter (cm) of the mortars with the yield stress is plotted in Figure 5.

**Figure 6:**
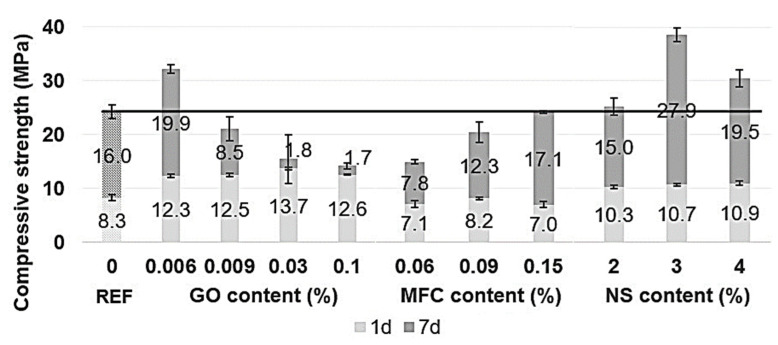
Compressive strength results at 1 and 7 days, according to GO, MFC and NS content (%). The numbers on each bar indicate the additional strength brought between 1 and 7 days.

The yield stress–spread straight calculated for the rheological parameter of the tests was plotted in Figure 6 with the equation τ_0_ = 1000.3^(−0.233 D)^. For the experimental data that do not follow this line, it can be assumed that the rheology is affected by the nanoadditives due to a change in viscosity. This effect is particularly marked for MFC. For these nanoadditives, the flow requires a higher yield stress; the regime change between viscous flow regimes and pseudo-viscous flow is supposed to be due to the water kept by MFC.

### 3.4. Mechanical Behaviour at an Early Age

**Figure 7:**
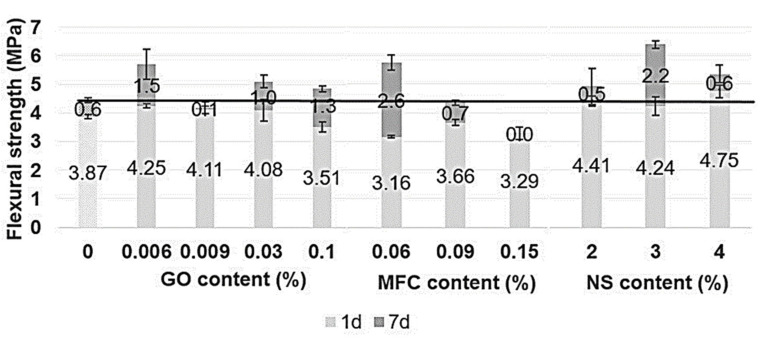
Flexural strength results at 1 and 7 days, according to GO, MFC and NS content (%).

Mechanical properties were tested at 1 and 7 days after mixing through compressive and flexural strength tests, as can be seen in Figure 6 and Figure 7, respectively.

It must be considered that this cement type V, with low clinker content (56%) and high additions (slag 22% and fly ash 22%), is known to reach full hydration in the long term (90 days). The characteristic compressive strength at 28 days is still lower than other types of cement. Therefore, the results on mechanical properties, both compression and flexural strength, should be considered as not fully developed and were performed to control the evolution and to compare between the different nanoadditives.

As shown in Figure 6, compressive strength depended on the type of nanoadditives used. GO, NS and MFC reacted differently to each other. Increasing the dosage allowed determining the optimal amount for each type of nanomaterial, although the effect on compressive and flexural might not correspond for the same dosage.

GO additions significantly improved both compressive and flexural strengths at the early age (1 day) compared with 7 days. However, the behaviour in compression and bending differs because increasing the amount of GO tended to increase the compression and decrease the bending. At 7 days, the enhancement in compression was only observed for the minor amounts of GO. Interestingly, GO addition did not show consistent results. After 7 days of curing, only GO 0.006 increased the compressive strength by 33%, while GO 0.009, GO 0.03 and GO 0.1 decreased the compressive strength by 13%, 36% and 41%, respectively.

On the other hand, the flexural strength was higher at 7 days for any amount of GO. A GO addition of 0.006% was found to be the optimal amount; an extra addition may have weakened the microstructure by creating nanoplatelets clusters that would not react with water and cement.

The effect of NS on mechanical enhancement was fast due to its high specific surface area and particle size. NS particles react quickly, providing amorphous silica for cement hydration products before serving as nucleation nodes for C-S-H gel. Any NS added to cement mortar increased compression strength at 24 h and at 7 days. The increase at 24 h was around 30% for any amount. At 7 days, the improvement varied a lot for the different amounts, observing the optimal behaviour for 3% of NS, resulting in an increase of 59%.

The effect of MFC on the mechanical properties is not highlighted as positive for any amount. At 1 day, both compressive and flexural strength decreased. MFC addition decreased the compressive strength for about 38%, 16% and 1% at 0.06%, 0.09% and 0.15%, respectively, at 7 days. Only MFC 0.06% enhanced the flexural strength. The reduction of workability suggests that less water was available to react with cement particles as microcellulose fibrils could have absorbed it.

### 3.5. Analysis of Nanoadditive Effect on the Studied Properties

We collected the main results from the different tests performed to assess the properties at the fresh state and hardening at an early age in Table 4. The effect of using nanoadditives can have linear, threshold or non-monotonic behaviour. The response of the nanoadditives is related to the nature of the effect in the studied properties. The size, form and composition modify the cement hydration reaction in different ways, and it can be measured on hardening and fresh state properties.

For the setting time test, the behaviour is linear or threshold. The setting time is considerably modified once the dosage reaches a threshold, and increasing the nanoadditive amount, the effect may be more pronounced. The exception is MFC, where the setting time decreases immediately with any amount. There is a linear relationship between dosage and setting time, both initial and final. The same behaviour is observed for workability. The effect of the MFC is related to the water absorbed by the fibres, which reduce the water available for the cement hydration. If this absorbed water allows further hydration, it may be detected by an improving in mechanical properties at 28 or 90 days.

The workability of the samples with the nanoadditives has a non-monotonic behaviour except for the MFC, where the workability is highly reduced. The GO samples, with low dosages, increase the workability at t_0_, but dosages over 0.03% reduce the workability. If the workability is an important criterion according to the final application, this property could be used to select the optimal dosage of GO to add.

The influence of all the studied nanoadditives on the mechanical properties has a non-monotonic behaviour. This allows selecting an optimal dosage for the nanoadditive, which is very interesting in terms of cost. The compression and flexural strength optimal dosage does not correspond to the same amount of nanoadditive. However, the most used criterion is to select the optimal dosage according to the performance of the compression test.

## 4. Conclusions

- Nanoadditives modify the hydration kinetics. At a mortar scale, it could be observed on the workability.
- GO: Influences the cement hydration reaction, leading to a reduction of the setting time. The workability increases at a low amount of GO, while at higher dosages, GO leads to an increasing reduction of the workability.
- MFC: Had a significant impact on the workability due to their interaction with mixing water and observed in the reduction of spread diameters in the slump test. The effect on mechanical properties was dose dependent. At higher dosages, the compressive strength increased; the result was more important at 7 days than at 1 day, which means a further hydration reaction with the water kept on the MFC. The absorption of water around its surroundings enhanced hydration, and consequently reduced the setting time.
- NS: It is highly reactive, and promotes and accelerates the cement particles’ hydration reaction due to its large specific surface. This was shown in isothermal calorimetry and setting time experiments.

## Figures and Tables

**Table 1:** Characteristics of cement V and nanoaddditives (GO, MCF, NS and superplasticiser) used in this work. C, O, S, H and N refers to the chemical names of Carbon, Oxygen, Sulphur, Hydrogen and Nitrogen, respectively. CaO is calcium oxide, Al_2_O_3_ is Aluminium oxide, Fe_2_O_3_ is Iron III oxide, and MgO is magnesium oxide.

**GRAPHENE OXIDE, GO**					
Chemical element	C	O	S	H	N
Mass weight (%)	49–56	41–50	2–4	0–1	0–1
**MICROFIBRILLATED CELLULOSE, MFC**					
Specific surface area (m^2^/g)	31–33	Nominal fibre width (nm)			50
**NANOSILICA, NS**					
Particles size (nm)	15	Loss of ignition after 2 h 1000 °C (%)			7
Specific surface area (m^2^/g)	140	pH (5 wt%, aqueous slurry)			4
**CEMENT CEM V/A**					
Chemical compound	CaO	SiO_2_	Al_2_O_3_	Fe_2_O_3_	MgO
Mass weight (%)	46.2	31.1	10.2	3.6	2.7
Blaine surface (m^2^/kg)	502.3	Hydration heat (J/g)		275 at 41 h, 309 at 120 h	
Initial setting time (min)	223	Consistency (water in cement paste)			29.50%
**AGGREGATES: SAND**					
Grain size (mm)	0/2	Density (at 20 °C, kg/m^3^)			2600
**SUPERPLASTICISER, SP**					
pH (aqueous solution)	3.7	Density (g/cm^3^)			1.06
Solid content (%)	40	Water content (%)			60

**Table 2:** Cement paste formulations.

Sample Name	Nano-Additive/Cement Ratio [% in Weight of Cement]	Nano-Additive [mg]	Mixing Water [g]
REF	-	-	176.0
GO 0.006	0.006	27	169.2
GO 0.009	0.009	40	165.9
GO 0.03	0.030	135	142.3
GO 0.1	0.100	450	63.9
MFC 0.06	0.060	270	162.7
MFC 0.09	0.090	405	156.1
MFC 0.15	0.150	675	142.9
NS 2	2.000	9·10^3^	176.0
NS 3	3.000	13.5 10^3^	176.0
NS 4	4.000	18 10^3^	176.0

**Table 3:** Influence of nanomaterials on the hydration heat characteristics. In brackets, the variations from the REF values (%). HF (q_min_) represents the minimum heat flow at induction period, t (q_min_) represents the duration of (initiation + induction) periods, HF (q_max_) represents the peak of heat flow, and t (q_max_) represents the time at sulphate depletion peak. We tested only one sample of each formulation for the isothermal calorimetry test that lasts 7 days.

Sample	HF (q_min_) [mW/g]	t (q_min_) [h]	t (q_max_) [h]	HF (q_max_) [mW/g]	Cumulative Heat [J/g]
REF	0.173	4.59	22.53	1.975	230.5
GO 0.006	0.162 (−6%)	4.73 (+3%)	22.92 (+2%)	1.981 (+1%)	228.8 (−1%)
GO 0.009	0.161 (−6%)	4.75 (+3%)	22.83 (+1%)	2.004 (+2%)	234.0 (+1%)
GO 0.1	0.163 (−6%)	4.67 (+2%)	2.00 (−2%)	1.982 (+1%)	230.5 (=)
NS 2	0.283 (+64%)	3.80 (−17%)	19.67 (−15%)	2.069 (+5%)	226.5 (−2%)
NS 3	0.388 (+124%)	3.77 (−18%)	19.34 (−16%)	2.129 (+7%)	242.3 (+5%)

**Table 4:** Summaries of the obtained results on performed tests.

Sample	Reference	GO [0.006–0.1%]	MFC [0.06–0.15%]	NS [2–4%]
**Setting time [min]**				
**Initial**	230	220–170	220–170	220–110
**Final**	260	260–210	190	230–130
**Isothermal calorimetry:**				
		Modification observed at high dosage and after deceleration period	Not measured	Heat flow increase and acceleration of the reaction
**Cumulative heat** **at 6 days**	230.5 J/g	228.8 J/g	234 J/g	230.5 J/g
**Workability: Non-monotonic or linear relation**				
		Non-monotonic effect: reduction and improvement of the workability	Linear reduction of the workability	Non-monotonic increase of the workability
**Spread diameter** **at t_0_**	30.7 cm	32.8–27.8 cm	26–14.7 cm	33–34 cm
**Mechanical properties: Non-monotonic or linear relation**				
**Compression strength** **at 7 days**	24.3 MPa	Optimal dosage 0.006% 32.3 MPa	Linear relation 0.15% 24.1 MPa	Optimal dosage 3% 38.6 MPa
**Flexural strength** **at 7 days**	3.87 MPa	Optimal dosage 0.006% 4.25 MPa	Optimal dosage 0.09% 3.66 MPa	Optimal dosage 4% 4.75 MPa

## Data Availability

Not applicable.

## References

[1] Lothenbach B., Scrivener K., Hooton R.D. (2011). Supplementary cementitious materials. Cem. Concr. Res..

[2] Li G.Y., Wang P.M., Zhao X. (2005). Mechanical behavior and microstructure of cement composites incorporating surface-treated multi-walled carbon nanotubes. Carbon.

[3] de Souza L.O., Cordazzo M., de Souza L.M.S., Tonoli G., Silva F.D.A., Mechtcherine V. (2021). Investigation of dispersion methodologies of microcrystalline and nano-fibrillated cellulose on cement pastes. Cem. Concr. Compos..

[4] Wu J., Ding Q., Yang W., Wang L., Wang H. (2021). Influence of Submicron Fibrillated Cellulose Fibers from Cotton on Hydration and Microstructure of Portland Cement Paste. Molecules.

[5] Barbhuiya G.H., Moiz M.A., Hasan S.D., Zaheer M.M. (2020). Effects of the nanosilica addition on cement concrete: A review. Mater. Today Proc..

[6] Björnström J., Martinelli A., Matic A., Börjesson L., Panas I. (2004). Accelerating effects of colloidal nano-silica for beneficial calcium–silicate–hydrate formation in cement. Chem. Phys. Lett..

[7] Dubey N., Rajan S.S., Bello Y.D., Min K.-S., Rosa V. (2017). Graphene Nanosheets to Improve Physico-Mechanical Properties of Bioactive Calcium Silicate Cements. Materials.

[8] Goracci G., Dolado S.J. (2020). Elucidation of Conduction Mechanism in Graphene Nanoplatelets (GNPs)/Cement Composite Using Dielectric Spectroscopy. Materials.

[9] Wu Y.-Y., Que L., Cui Z., Lambert P. (2019). Physical Properties of Concrete Containing Graphene Oxide Nanosheets. Materials.

[10] Galao O., Baeza F.J., Zornoza E., Garcés P. (2017). Carbon Nanofiber Cement Sensors to Detect Strain and Damage of Concrete Specimens Under Compression. Nanomaterials.

[11] Vegas I., Gaitero J., Urreta J., García R., Frías M. (2014). Aging and durability of ternary cements containing fly ash and activated paper sludge. Constr. Build. Mater..

[12] Giménez R.G., De La Villa R.V., Rubio V., Vegas I., Frías M. (2015). Forced Aging and Ionic Mobility of Ternary Cements Exposed to Aggressive Saline Marine Environments and Cryoclastic Processes. Water Air Soil Pollut..

[13] Alaghebandian N., Mirvalad S., Javid A.A.S. (2020). Durability of self-consolidating concrete and mortar mixtures containing ternary and quaternary cement blends exposed to simulated marine environment. Constr. Build. Mater..

[14] Thomas M., Shehata M., Shashiprakash S., Hopkins D., Cail K. (1999). Use of ternary cementitious systems containing silica fume and fly ash in concrete. Cem. Concr. Res..

[15] De Weerdt K., Ben Haha M., LE Saout G., Kjellsen K., Justnes H., Lothenbach B. (2011). Hydration mechanisms of ternary Portland cements containing limestone powder and fly ash. Cem. Concr. Res..

[16] Dodson V.H. (2013). Concrete Admixtures.

[17] Sant G., Lothenbach B., Juilland P., LE Saout G., Weiss W., Scrivener K. (2011). The origin of early age expansions induced in cementitious materials containing shrinkage reducing admixtures. Cem. Concr. Res..

[18] Zhu W., Bartos P.J., Porro A. (2004). Application of nanotechnology in construction. Mater. Struct..

[19] Long W.-J., Zheng D., Duan H.-B., Han N., Xing F. (2018). Performance enhancement and environmental impact of cement composites containing graphene oxide with recycled fine aggregates. J. Clean. Prod..

[20] Reches Y. (2018). Nanoparticles as concrete additives: Review and perspectives. Constr. Build. Mater..

[21] Cao Y., Zavaterri P., Youngblood J., Moon R., Weiss W. (2015). The influence of cellulose nanocrystal additions on the performance of cement paste. Cem. Concr. Compos..

[22] Sanchez F., Sobolev K. (2010). Nanotechnology in concrete—A review. Constr. Build. Mater..

[23] Mohammed A., Sanjayan J., Duan W.H., Nazari A. (2015). Incorporating graphene oxide in cement composites: A study of transport properties. Constr. Build. Mater..

[24] Hawreen A., Bogas J.A. (2018). Influence of carbon nanotubes on steel–concrete bond strength. Mater. Struct..

[25] Chuah S., Pan Z., Sanjayan J., Wang C., Duan W.H. (2014). Nano reinforced cement and concrete composites and new perspective from graphene oxide. Constr. Build. Mater..

[26] Arshad A., Jabbal M., Yan Y., Reay D. (2019). A review on graphene based nanofluids: Preparation, characterization and applications. J. Mol. Liq..

[27] Hou D., Lu Z., Li X., Ma H., Li Z. (2017). Reactive molecular dynamics and experimental study of graphene-cement composites: Structure, dynamics and reinforcement mechanisms. Carbon.

[28] Ho V.D., Ng C.-T., Ozbakkaloglu T., Goodwin A., McGuckin C., Karunagaran R.U., Losic D. (2020). Influence of pristine graphene particle sizes on physicochemical, microstructural and mechanical properties of Portland cement mortars. Constr. Build. Mater..

[29] Wang Y., Yang J., Ouyang D. (2019). Effect of Graphene Oxide on Mechanical Properties of Cement Mortar and its Strengthening Mechanism. Materials.

[30] Ghazizadeh S., Duffour P., Skipper N.T., Bai Y. (2018). Understanding the behaviour of graphene oxide in Portland cement paste. Cem. Concr. Res..

[31] Lu Z., Hanif A., Ning C., Shao H., Yin R., Li Z. (2017). Steric stabilization of graphene oxide in alkaline cementitious solutions: Mechanical enhancement of cement composite. Mater. Des..

[32] Yang H., Monasterio M., Cui H., Han N. (2017). Experimental study of the effects of graphene oxide on microstructure and properties of cement paste composite. Compos. Part A Appl. Sci. Manuf..

[33] Du H., Gao H.J., Pang S.D. (2016). Improvement in concrete resistance against water and chloride ingress by adding graphene nanoplatelet. Cem. Concr. Res..

[34] Du H., Pang S.D. (2015). Enhancement of barrier properties of cement mortar with graphene nanoplatelet. Cem. Concr. Res..

[35] Pei C., Ueda T., Zhu J. (2020). Investigation of the effectiveness of graphene/polyvinyl alcohol on the mechanical and electrical properties of cement composites. Mater. Struct..

[36] Yang H., Cui H., Tang W., Li Z., Han N., Xing F. (2017). A critical review on research progress of graphene/cement based composites. Compos. Part A Appl. Sci. Manuf..

[37] Fu T., Moon R.J., Zavattieri P., Youngblood J., Weiss W.J., Jawaid M., Boufi S., Abdul Khalil H.P.S. (2017). Cellulose nanomaterials as additives for cementitious materials. Cellulose-Reinforced Nanofibre Composites.

[38] Kargarzadeh H., Mariano M., Gopakumar D., Ahmad I., Thomas S., Dufresne A., Huang J., Lin N. (2018). Advances in cellulose nanomaterials. Cellulose.

[39] Kolour H.H., Ashraf W., Landis E.N. (2020). Hydration and Early Age Properties of Cement Pastes Modified with Cellulose Nanofibrils. Transp. Res. Rec. J. Transp. Res. Board.

[40] Aitcin P. (2003). The durability characteristics of high performance concrete: A review. Cem. Concr. Compos..

[41] Monasterio M., Gaitero J.J., Erkizia E., Bustos A.G., Miccio L.A., Dolado J.S., Cerveny S. (2015). Effect of addition of silica- and amine functionalized silica-nanoparticles on the microstructure of calcium silicate hydrate (C–S–H) gel. J. Colloid Interface Sci..

[42] Gaitero J., Campillo I., Guerrero A. (2008). Reduction of the calcium leaching rate of cement paste by addition of silica nanoparticles. Cem. Concr. Res..

[43] Liu X., Feng P., Shu X., Ran Q. (2019). Effects of highly dispersed nano-SiO_2_ on the microstructure development of cement pastes. Mater. Struct..

[44] Jo B.-W., Kim C.-H., Tae G.-H., Park J.-B. (2007). Characteristics of cement mortar with nano-SiO_2_ particles. Constr. Build. Mater..

[45] Land G., Stephan D. (2015). Controlling cement hydration with nanoparticles. Cem. Concr. Compos..

[46] (2020). NF EN 196-3-Septembre 2017. AFNOR, S.A.N. https://www.boutique.afnor.org/norme/nf-en-196-3/methodes-d-essai-des-ciments-partie-3-determination-du-temps-de-prise-et-de-la-stabilite/article/904098/fa186374.

[47] NF EN 196-1-Septembre 2016. https://www.boutique.afnor.org/norme/nf-en-196-1/methodes-d-essais-des-ciments-partie-1-determination-des-resistances/article/866862/fa184622.

[48] NF EN 196-11-December 2018. https://www.boutique.afnor.org/norme/pr-nf-en-196-11/methods-of-testing-cement-part-11-heat-of-hydration-isothermal-conduction-calorimetry-method/article/905598/fa189490.

[49] Roussel N., Stefani C., Leroy R. (2005). From mini-cone test to Abrams cone test: Measurement of cement-based materials yield stress using slump tests. Cem. Concr. Res..

[50] Kong D., Huang S., Corr D., Yang Y., Shah S.P. (2018). Whether do nano-particles act as nucleation sites for C-S-H gel growth during cement hydration?. Cem. Concr. Compos..

[51] Abbasi S., Jannaty M.H., Faraj R.H., ShahbazPanahi S., Mosavi A. (2020). The Effect of Incorporating Silica Stone Waste on the Mechanical Properties of Sustainable Concretes. Materials.

[52] Karen Scrivener R.S., Lothenbach B. (2017). A Practical Guide to Microstructural Analysis of Cementitious Materials.

